# Socioeconomic disparities in preventable hospitalization among adults with diabetes in Taiwan: a multilevel modelling approach

**DOI:** 10.1186/s12939-015-0160-4

**Published:** 2015-03-21

**Authors:** Pei-Ching Chen, Ching-Yao Tsai, Lin-Chung Woung, Yue-Chune Lee

**Affiliations:** Institute of Health and Welfare Policy, National Yang-Ming University, No.155, Sec. 2, Linong St, Beitou Dist, Taipei City, 112 Taiwan; Department of Education and Research, Taipei City Hospital, No.145, Zhengzhou Rd, Datong Dist, Taipei City, 103 Taiwan; Department of Ophthalmology, Zhongxing Branch, Taipei City Hospital, No.145, Zhengzhou Rd, Datong Dist, Taipei City, 103 Taiwan; Institute of Public Health, National Yang-Ming University, No.155, Sec. 2, Linong St, Beitou Dist, Taipei City, 112 Taiwan; Institute of Hospital and Health Care Administration, National Yang-Ming University, No.155, Sec. 2, Linong St, Beitou Dist, Taipei City, 112 Taiwan

**Keywords:** Socioeconomic, Disparities, Diabetes, Preventable hospitalizations

## Abstract

**Introduction:**

Literature shows socioeconomic disparities are related to various aspects of diabetes care. However, few studies have explored the relationship between socioeconomics and healthcare outcomes, particularly with regard to preventable hospitalization. This cohort study employed hierarchical modelling to evaluate the role of socioeconomics at both the individual and regional levels in order to examine disparities associated with the preventable hospitalization of diabetes patients in Taiwan.

**Methods:**

This study employed the Longitudinal Health Insurance Database 2010, which provided a representative cohort comprising one million people enrolled in Taiwan’s National Health Insurance in 2010. All diabetes patients aged 18 and older who received regular care in 2010 were included in this study. The outcome examined in this study was diabetes-related preventable hospitalization during the period of 2010 to 2011. Socioeconomic status at the individual level was measured according to income and at the regional level according to level of urbanization and the proportion of residents who had completed college education. Control variables included age, gender, comorbidities, time of diabetes diagnosis, participated in the pay-for-performance program status, and the characteristics of regular sources of care, including the level of the facility (i.e., medical centre, regional hospital, local hospital, outpatient clinic) and ownership. Statistical analysis was performed using generalized linear mixed models.

**Results:**

A total of 57,791 patients from 25 regions diagnosed with type-2 diabetes mellitus were identified in the National Health Insurance claim data for the year 2010. 1040 of these patients (1.8%) had at least one diabetes-related preventable hospitalization event during the period of 2010–2011. After controlling for the characteristics of patients and health care providers, our results show that dependents and patients in low and middle income brackets (OR = 2.48, 2.44, and 2.08 respectively) as well as those living in regions with a low, median, or high education bracket (OR = 1.32, 1.38, and 1.46 respectively) face a higher probability of preventable hospitalization.

**Conclusions:**

Our results demonstrate that the socioeconomic effects of higher education at the regional level as well as income at the individual level are important factors which affect disparities in diabetes-related preventable hospitalization.

## Main text

### Background

Diabetes mellitus is common in industrialized nations; however, it disproportionately affects adults on the lower end of the socioeconomic scale. Most previous studies that examined socioeconomic disparities related to the incidence or prevalence of diabetes [1-10] have found that individuals with a low income [1-3,5,6,9], lower education [2,6,9], a blue-collar occupation [7], as well as those living in disadvantaged areas [4,10] face a higher probability of contracting diabetes. Economically and educationally disadvantaged regions were also found to exhibit a higher prevalence of the disease.

Many studies have examined socioeconomic disparities related to various aspects of diabetes care, such as the method of treatment [1,2], the quality of care [11-16], and health outcomes such as mortality and death [17-20]. Results from previous research indicates that individuals with a higher socioeconomic status are more likely to be tested or treated for diabetes [1,2,4,16], more likely to have normal glucose levels, normal blood pressure, and better cholesterol regulation [11-14,21], as well as a lower probability of diabetes-related complications [15] and mortality [17-20].

Few studies have examined the relationship between socioeconomic disparities and preventable hospitalization [22-24]. Nonetheless, an inverse relationship has been observed between income level and hospitalization rates. Indeed, previous researchers determined that living in rural areas [22] or low-income neighborhoods [22-24] was associated with preventable hospitalization. However, few researchers have focused on diabetes patients when exploring these relationships, and data related to socioeconomic status and preventable diabetes hospitalizations is particularly lacking at the regional and individual levels. The present cohort study used hierarchical modelling to evaluate the role of socioeconomic disparity at both individual and regional levels in cases of preventable diabetes hospitalizations in Taiwan.

## Methods

The research involved a cohort study of diabetes patients. Subjects were followed for one year (2010) to determine whether they were admitted to hospital and whether these admissions were preventable.

### Data sources

This study employed the Longitudinal Health Insurance Database 2010 (LHID2010), in which the National Health Research Institute (NHRI) randomly sampled a representative cohort comprising one million of the 23.16 million individuals enrolled in the National Health Insurance program in 2010. The study collected data related to ambulatory care, inpatient expenditures, and registration for all patients (both inpatients and outpatients) included in the study. All case IDs required for data linkage were encrypted prior to release in order to protect the privacy of the individuals. The data used in this study does not possess any unique patient identifiers or any sensitive information that could be traced back to individual patients. No significant differences related to gender, age, or average insurance contribution were observed between patients in the LHID 2010 and the overall population [25].

### Study cohorts

The study population included patients diagnosed with diabetes mellitus in 2010. Patients were defined as having diabetes mellitus if one of the following criteria were met: (1) the patient was diagnosed under the *International Classification of Diseases, Ninth Revision, Clinical Modification* (*ICD-9-CM*) with a code of 250.0 – 250.9 following an ambulatory visit; or (2) the patient was diagnosed with an ICD-9-CM code of 250.x following an inpatient visit. Validation of the ICD coding for diabetes mellitus required that patients be diagnosed at least twice by a physician within one year or have been hospitalized once under relevant diabetes ICD codes [26]. A total of 57,791 patients from 25 regions diagnosed with type-2 diabetes mellitus were identified from LHID2010 in 2010. Each region included between 152 and 9,938 patients with the exception of Lienchiang County, which had only 24 patients. These patients were divided into two categories: (1) new cases in 2010 and (2) previous diabetes patients, based on diabetes status in 2009.

### Measure of socioeconomic status

Socioeconomic status was assessed at the individual and regional level. Income was used as an indicator of socioeconomic status at the individual level. The income category was divided into four groups according to employment status and monthly salary: family-dependent (monthly salary was 0), low (included low-income households, veterans, and unemployed individuals), middle (monthly salary < NTD 30,000), and high (monthly salary ≥ NTD 30,000).

Regional data was obtained from all 25 counties of Taiwan for the year 2010. To evaluate socioeconomic status at the regional level, we considered tertiary education and level of urbanization. The ratio of individuals with a tertiary education was defined as the proportion of individuals who attended colleges or universities within a given region, which ranged from 18.68% to 60.94%. These values were divided into four categories (highest, high, median, and low) according to quartile values obtained in various regions of Taiwan (Q1 < 26.89, Q2 = 26.89-28.45, Q3 = 28.46-36.99, Q4 > 36.99). Level of urbanization was classified as either urban (Taipei City and Kaohsiung City) or rural.

### Outcome measure

The need for hospitalization can be avoided by providing effective outpatient care. In this study, the number of preventable hospitalizations was determined using data related to hospital admissions for ambulatory care sensitive conditions (ACSCs). Specifically, this data was used to identify potential barriers to ambulatory care, assess the performance of the primary care delivery system, and identify possible deficiencies in the quality of outpatient care [27].

This study adopted the definition of hospitalization from the Prevention Quality Indicator (PQI) algorithm proposed by the Agency for Healthcare Research and Quality (AHRQ; [28]). Specifically, we selected four indicators relevant to diabetes: short-term and long-term complications associated with diabetes upon admission, uncontrolled diabetes upon admission, and lower-extremity amputation among diabetes patients. Using data related to inpatient expenditures, we selected all cases where patients met the following criteria: they were 18 years or older; they had been discharged from a hospital; and they had (1) short-term complications which were assigned ICD-9-CM principal diagnosis codes for diabetes with ketoacidosis (250.1), hyperosmolarity (250.2), or coma (250.3); (2) long-term complications assigned ICD-9-CM codes for diabetes with renal (250.4), eye (250.5), neurological (250.6), circulatory (250.7), or other unspecified complications (250.8-250.9); or (3) uncontrolled diabetes upon admission without mention of any short- or long-term complications (250.0). We also included patients who had been assigned (4) ICD-9-CM procedure codes for lower-extremity amputations in any field and diagnosis codes for diabetes in any field. We excluded pregnant patients, those that had recently given birth, and those who had been transferred from another institution.

We adopted the first diagnosis of diabetes in 2010 as the index time and followed patients for one year to determine whether they were admitted to hospital and whether these admissions were cases of preventable diabetes-related hospitalization. We respectively counted the frequency of each type of diabetes-related preventable hospitalization. If the patient had at least one case of each preventable hospitalization then the outcome was labelled as yes (Y = 1) while others was labelled as no (Y = 0).

### Control variables

Control variables included the characteristics of patients and health care providers in 2010. Patient characteristics included sex, age, time of diabetes diagnosis (i.e. in 2010 or prior to 2010), participated in the P4P program status, and comorbidities one year prior to the index date (i.e. the first diagnosis of diabetes in 2010). This study adopted the 30 comorbidity measures developed by Elixhauser et al. [29].

In November 2001, the National Health Insurance Administration in Taiwan implemented Pay-for-Performance (P4P) programs for diabetes mellitus. P4P is an innovative payment system for health insurance, in which financial incentives are provided to health providers with the aim of improving the quality of care [30,31]. Previous studies have reported positive results from diabetes P4P programs in Taiwan [32,33]; thus, enrolment in the P4P program was included as an independent variable in our analysis.

The characteristics of regular health care providers included the level of the facility (medical center, regional hospital, local hospital, or clinic) and ownership (public or private). Patients in Taiwan are given the freedom to select their health care providers and are also entitled to use more than one provider. To overcome complications associated with patients who made multiple visits to different providers, patients were assigned to the health care provider who took on more than half of visits in a given year.

### Statistical analyses

Generalized linear mixed models with a binary distributed response and a random intercept term for the region were used to examine the relationships between socioeconomic factors and the outcome (preventable hospitalization), while accounting for the clustering of patients within regions. Fixed-effect slopes were examined for individual- and regional-level independent variables. Odds ratios (OR) and 95% confidence intervals (CI) were reported for each variable in multiple hierarchical logistic regressions. Models were fitted using the GLIMMIX procedure in SAS 9.2.

## Results

### Characteristics of diabetes patients

Table 1 presents the characteristics of diabetes patients aged 18 years and older in 2010. Among these, 50.70% were male, with a mean age of at least 62.59 years; 33.46% earned a middle-level income; 71.74% possessed at least one comorbidity; 18.10% were new diabetes patients in 2010; 19.29% joined in the P4P program; 75.07% received treatment from private health care providers; and 31.58% received treatment in clinics.

**Table 1:** **Characteristics at individual and regional levels among diabetes patients aged 18 years or older in 2010**

**Variables (n = 57,791)**	**% or Mean(SD)**
*Individual-level variables*	
Gender	
Male	50.70
Female	49.30
Age *(Mean (SD))*	62.59 (13.07)
<55	26.69
55-65	29.09
65-75	24.15
≥75	20.06
Income	
Dependents	32.43
Low (low-income households, veterans, and unemployed)	16.90
Middle (< NTD 30,000)	33.46
High ( ≥ NTD 30,000)	17.21
Time of diabetes diagnosis	
New patients in 2010	18.10
Previous patients	81.90
Comorbidities	
0	28.26
1	41.04
≥2	30.7
Participated in the P4P program	
Yes	19.29
No	80.71
Receives regular care from a health care professional	
Yes	90.11
No	9.89
Health care provider ownership (n = 52,074)	
Public	24.93
Private	75.07
Health care provider level (n = 52,074)	
Medical Center	23.23
Regional Hospital	29.58
Local Hospital	15.60
Clinic	31.58
*Regional-level variables*	
Ratio of individuals with a higher education	
Highest	47.75
High	18.27
Median	20.00
Low	13.98
Level of urbanization	
Urban	24.16
Rural	75.84

Regional-level variables included the ratio of individuals with higher education and individuals living in urban vs. rural environments for the year 2010. The average ratio of tertiary educated individuals was 38.17%, and the range was 18.68% to 60.94%. According to the quartile values obtained in various regions of Taiwan (Q1 < 26.89, Q2 = 26.89-28.45, Q3 = 28.46-36.99, Q4 > 36.99), the ratio of individuals with tertiary education was divided into four categories: highest (47.75%), high (18.27%), median (20.00%) and low (13.98%). Most diabetes patients lived in regions with the highest average education (47.75%) or in rural regions (75.84%).

Table 2 presents the cases of preventable hospitalizations among diabetes patients during 2010–2011. Cases of preventable hospitalization included those related to short-term (224) and long-term (702) diabetes-related complications, uncontrolled diabetes upon admission (256), and lower-extremity amputations (116). The proportions of preventable hospitalizations included short-term (0.39%) and long-term (1.21%) diabetes-related complications upon admission, uncontrolled diabetes upon admission (0.44%), and lower-extremity amputation (0.20%). The proportions of specific types of preventable hospitalization were low; therefore, we combined them into a single analytic dependent variable (preventable hospitalization: 1.8%, n = 1040). In other words, 1040 of these patients had at least one diabetes-related preventable hospitalization event during the period of 2010–2011.

**Table 2:** **Cases of preventable hospitalization among diabetes patients aged 18 years or older during 2010-2011**

***Variables (n = 57,791)***	**n**	**%**
Diabetes-related preventable hospitalization		
Short-term complications upon admission	224	0.39
Long-term complications upon admission	702	1.21
Uncontrolled diabetes upon admission	256	0.44
Lower-extremity amputation	116	0.20
Any type of admission	1040	1.80

### Results of multilevel logistic regressions

Table 3 illustrates the differences in multilevel logistic regression results related to cases of preventable hospitalization among diabetes patients. Patients were separated into two models: the entire sample population (n = 57,791), and patients with regular health care providers (n = 52,074). Model 1 tested variable effects at both the individual and regional levels. Model 2 included the characteristics of health care providers. According to the model fit statistics, Model 2 provided a better model fit than Model 1.

**Table 3:** **Multilevel logistic regressions of preventable hospitalization among diabetes patients aged 18 years or older in 2010**

**Variables**	**Model 1**			**Model 2**		
	**OR**	**95% CI**		**OR**	**95% CI**	
*Individual level*						
Gender						
Male	1.027	0.905	1.166	1.038	0.903	1.194
Female	1.000			1.000		
Age						
<55	0.966	0.806	1.158	1.036	0.847	1.267
55-65	0.602^‡^	0.501	0.723	0.657^‡^	0.536	0.805
65-75	0.721^‡^	0.609	0.854	0.754^†^	0.624	0.911
≥75	1.000			1.000		
Income						
Dependents	2.657^‡^	2.021	3.493	2.475^‡^	1.854	3.303
Low	2.892^‡^	2.186	3.827	2.443^‡^	1.812	3.295
Middle	2.092^‡^	1.598	2.738	2.079^‡^	1.568	2.758
High	1.000			1.000		
Time of diabetes diagnosis						
New patients in 2010	0.759^†^	0.637	0.905	0.887	0.732	1.076
Previous patients	1.000			1.000		
Comorbidities						
0	0.647^‡^	0.547	0.765	0.704^‡^	0.585	0.846
1	0.574^‡^	0.497	0.664	0.588^‡^	0.501	0.690
≥2	1.000			1.000		
Participated in the P4P program						
Yes	0.704^‡^	0.589	0.841	0.640^‡^	0.528	0.776
No	1.000			1.000		
HCP Ownership						
Public				1.005	0.861	1.172
Private				1.000		
HCP Level						
Medical center				3.514^‡^	2.709	4.558
Regional hospital				4.917^‡^	3.867	6.254
Local hospital				3.818^‡^	2.929	4.977
Clinic				1.000		
*Regional level*						
Ratio of individuals with a higher education						
Highest	1.000			1.000		
High	1.541^‡^	1.257	1.889	1.463^†^	1.163	1.84
Median	1.328^†^	1.097	1.608	1.380^†^	1.116	1.706
Low	1.330^†^	1.099	1.610	1.316*	1.065	1.626
Level of urbanization						
Urban	0.837	0.686	1.022	0.826	0.665	1.026
Rural	1.000			1.000		
−2 Log likelihood (random effects)	10116.81			8253.20		

Model 1 controlled for characteristics at the individual and regional levels.

Model 2 controlled for model 1 variables and characteristics of the regular health care provider.

*P < 0.05, ^†^P < 0.01, ^‡^P < 0.001.

In the null model, significant variation was observed in the log odds of preventable hospitalization across the 25 counties of Taiwan (p <0.001). This result revealed evidence of regional effects.

As shown in Model 1 (in Table 3), individual income was associated with a significant decrease in the log odds of preventable hospitalization, indicating that patients in low and middle income brackets and those with a dependence on family members were more likely to undergo preventable hospitalization than those with a higher income. The odds ratios were 2.89, 2.09, and 2.66, respectively, with a p-value of less than 0.001. The effect size was moderate when the odds ratio exceeded 2.

In addition, regional education was significantly associated with a decrease in the log odds of preventable hospitalization, indicating that individuals living in regions with low, median, or high education brackets were more likely to undergo preventable hospitalization than those living in regions with the highest education level. The odds ratios of regional education were 1.33, 1.33, and 1.54, respectively. The effect size was small when the odds ratio exceeded 1.5.

Compared to patients over 75 years of age, those aged 55 to 75 were less likely to face preventable hospitalization. We obtained the same result for patients with zero or one comorbidity (compared to those with two comorbidities), patients who joined the P4P program (compared to those who did not join), and patients newly diagnosed with diabetes (compared to those with a previous diabetes diagnosis).

Model 2 supports the findings of Model 1, showing that the log odds of preventable hospitalization were significantly associated with socioeconomic status at both the individual and regional levels.

Similarly, after controlling for characteristics of patients and health care providers, patients living in regions with low, median, or high education brackets were more likely to undergo preventable hospitalization than those living in regions with the highest education level. For this, the odds ratios were 1.32, 1.38, and 1.46, respectively. The effect size was small when the odds ratio exceeded 1.5.

Furthermore, dependents and patients in the low or middle income bracket had a higher probability of undergoing preventable hospitalization than did those with a high income. The odds ratios were 2.48, 2.44, and 2.08, respectively, with a p-value of less than 0.001. The effect size was moderate when the odds ratio exceeded 2.

Patients receiving regular treatment in hospitals had significantly higher likelihood of preventable hospitalization compared to patients receiving regular treatment in clinics.

## Discussion

This research provides evidence that socioeconomic status at both the individual and regional levels is an important factor associated with disparities among cases of diabetes-related preventable hospitalization in Taiwan. Our results show that the socioeconomic effects of higher education (at the regional level) as well as income (at the individual level) were important factors. Patients in the lower income bracket as well as those living in regions with a large number of individuals with lower education had a higher probability of undergoing preventable hospitalization.

Our results demonstrate that individuals with a lower education and/or lower income were at higher risk of diabetes-related preventable hospitalization. These results echo the term inequity as identified by Whitehead [34], which refers to differences in health status which are unnecessary or avoidable and also considered unjust. For example, people with a low income or those living in disadvantaged areas are more likely to be exposed to living and working conditions that are unhealthy or stressful. Similarly, they are more likely to have inadequate access to essential healthcare and other public services. These differences could therefore be classified as inequities in healthcare.

Our results are also in agreement with findings presented by Brown et al. [35], who proposed a conceptual framework to illustrate the relationship between socioeconomic position (SEP) and health among individuals with diabetes mellitus. Specifically, one of the three pathways proposed by Brown to describe the relationship between SEP and health outcomes among diabetes patients was observed in our study. In short, individuals with low SEP (as measured by individual or household income), and/or those living in less privileged areas were more likely to face poorer health outcomes.

This was the first study to use multilevel modelling to explore the relationship between socioeconomic status and preventable hospitalizations. Previous studies [22-24] have used regional income for differentiation; however, no previous study has divided income level at the individual level. Our findings support previous evidence which indicated that a lower income is related to lower quality of care [11-14,21]. Thus, we can deduce that failure to obtain effective care may result in preventable hospitalization. Diabetes patients in the low or middle income bracket (including dependents) had a higher probability of preventable hospitalization, even though those with a low income obtained medical co-payment waivers and a subsistence allowance from the government. This implies that the subsidy did not change or reduce disparities in preventable hospitalization. The policy implication is a need for more comprehensive primary care. One example of this is based on the fact that the P4P program decreased the probability of preventable hospitalization; therefore, educating patients to care for themselves may be one way to reduce these types of disparities.

Diabetes patients living in regions with a high proportion of well-educated individuals were significantly less likely to undergo preventable hospitalization. The same correlation was observed for individuals living in urban areas; however, the differences for individuals living in urban areas were not significant. These results differ from those of previous studies [22-24]. Based on neighborhood income level, Kim et al. [22] determined that rural areas and lower income neighborhoods were positively associated with preventable hospitalization. Using a regionally based income index for individuals, Agabiti et al. [23] determined that patients in lower income areas had a higher ACSC hospitalization rate. Based on neighborhood level income, Booth and Hux [24] determined that individuals in the lowest income bracket were more likely to be hospitalized or visit an emergency department compared to those in the highest quintile. These findings may be explained by the fact that the previous study did not consider the effects of socioeconomic status at both the regional and individual level. Our findings indicate that when the effect of income is considered at the individual level, the effect of urbanization at the regional level is eliminated.

Furthermore, this study examined socioeconomic status from the perspective of education as well as urbanization (as a proxy for income) at the regional level. Our results show that the effect of education appears to be more important than urbanization with regard to diabetes care. Our findings are similar to those obtained in previous studies that considered the effects of both education and income on diabetes care. Previous findings indicated that individuals with a higher education were more likely to receive treatment [2], more likely to control glucose levels [13], and more likely to receive better quality of care [16]. These findings may be explained by individual income effects eliminating effects of regional urbanization. They may also explain why individuals in regions with a higher average education had a significantly lower probability of preventable hospitalization but individuals in urban regions did not.

Several limitations of this study should be noted. First, information related to individual education was unavailable in the claims database of the National Health Insurance program. If we had been able to consider the influence of this factor, the effects of income at the individual level and education at the regional level may have decreased. Second, income was determined using monthly salary-based figures and employment data, while the National Health Insurance Administration has established groups according to premiums. As a result, the income values used to evaluate socioeconomic status are not necessarily sensitive to variations in income status. Furthermore, income and employment status were collinear; therefore, we only included income as a factor in the model. Future researchers could develop a more accurate means of measuring socioeconomic status. Finally, the time period covered in this study was brief, allowing for the observation of only a few cases of each type of preventable hospitalization; we therefore combined these types into a single analytic dependent variable. Future studies could conduct studies over longer follow-up durations and provide separate analysis for each of the four outcomes of preventable hospitalization. In spite of these limitations, this study provides solid empirical evidence to support the existence of two-level socioeconomic disparities in preventable hospitalizations associated with diabetes.

## Conclusions

Our results demonstrate that socioeconomic status at the individual and regional levels are important factors associated with disparities among cases of diabetes-related preventable hospitalizations in Taiwan. We recommend that the government as well as health care providers focus on the continuity of care for low income or otherwise vulnerable diabetes patients in order to prevent unnecessary hospitalizations.
